# Diazoxide affects mitochondrial bioenergetics by the opening of mKATP channel on submicromolar scale

**DOI:** 10.1186/s12860-020-00275-0

**Published:** 2020-04-19

**Authors:** Olga Akopova, Liudmila Kolchinskaya, Valentina Nosar, Iryna Mankovska, Vadim Sagach

**Affiliations:** 1 Circulation department, Bogomoletz Institute of Physiology, NAS of Ukraine, Bogomoletz str. 4, Kiev, 01601 Ukraine; 2 Hypoxic States Research Department, Bogomoletz Institute of Physiology, NAS of Ukraine, Kiev, Ukraine

**Keywords:** Diazoxide, Potassium transport, mK_ATP_-channel, Potassium cycle, Mild uncoupling, ROS production

## Abstract

**Background:**

Cytoprotection afforded by mitochondrial ATP-sensitive K^+^-channel (mK_ATP_-channel) opener diazoxide (DZ) largely depends on the activation of potassium cycle with eventual modulation of mitochondrial functions and ROS production. However, generally these effects were studied in the presence of Mg∙ATP known to block K^+^ transport. Thus, the purpose of our work was the estimation of DZ effects on K^+^ transport, K^+^ cycle and ROS production in rat liver mitochondria in the absence of Mg∙ATP.

**Results:**

Without Mg·ATP, full activation of native mK_ATP_-channel, accompanied by the increase in ATP-insensitive K^+^ uptake, activation of K^+^-cycle and respiratory uncoupling, was reached at ≤0.5 μM of DZ,. Higher diazoxide concentrations augmented ATP-insensitive K^+^ uptake, but not mK_ATP_-channel activity. mK_ATP_-channel was blocked by Mg·ATP, reactivated by DZ, and repeatedly blocked by mK_ATP_-channel blockers glibenclamide and 5-hydroxydecanoate, whereas ATP-insensitive potassium transport was blocked by Mg^2+^ and was not restored by DZ. High sensitivity of potassium transport to DZ in native mitochondria resulted in suppression of mitochondrial ROS production caused by the activation of K^+^-cycle on sub-micromolar scale. Based on the oxygen consumption study, the share of mK_ATP_-channel in respiratory uncoupling by DZ was found.

**Conclusions:**

The study of mK_ATP_-channel activation by diazoxide in the absence of MgATP discloses novel, not described earlier, aspects of mK_ATP_-channel interaction with this drug. High sensitivity of mK_ATP_-channel to DZ results in the modulation of mitochondrial functions and ROS production by DZ on sub-micromolar concentration scale. Our experiments led us to the hypothesis that under the conditions marked by ATP deficiency affinity of mK_ATP_-channel to DZ can increase, which might contribute to the high effectiveness of this drug in cardio- and neuroprotection.

## Background

Cytoprotective effects afforded by the mitochondrial K_ATP_-channel (mK_ATP_-channel) opening generally are supposed to result from the modulation of mitochondrial functions and protective redox signaling triggered by ATP-sensitive K^+^ transport, which helped tissues recovery from the impairments caused by ischemic, hypoxic [1–5] and metabolic stress conditions [6–9]. Numerous pathophysiological conditions primarily affect mitochondrial bioenergetic. Prevention of Ca^2+^ overload [8], restoration of ATP synthesis, prevention of mitochondrial depolarization [3] and the regulation of mitochondrial ROS production resulting from mK_ATP_-channels opening in different cell types were shown to block apoptotic and necrotic pathways triggered by multiple pathophysiological states [1–9].

The disclosure of the mechanisms underlying the modulation of mitochondrial functions by mK_ATP_-channels openers (pinacidil, diazoxide, nicorandil) needs the study of the direct effects of these drugs on ATP-sensitive potassium transport in mitochondria and their consequences for mitochondrial functions. However, their appraisal at present is complicated because of several off-target effects of pharmacological mK_ATP_-channel openers [10, 11].

Of the known mK_ATP_-channel openers diazoxide is most widely used one. Apparent activation constants for diazoxide show its much greater affinity to mitochondrial than plasma membrane, K_ATP_-channel [12]. Consistent with the present views, bioenergetic effects of diazoxide and its impact on ROS production are based on “mild uncoupling” of the respiratory chain due to the opening of mK_ATP_-channel and the activation of mitochondrial potassium cycle [13, 14]. But similar to other drugs, many side effects of diazoxide were described, not caused directly by mK_ATP_-channel opening and capable of affecting ROS production in a similar way, such as protonophoric properties at high micromolar concentrations [15], suppression of SDH (Complex II) activity and respiratory inhibition [16]. Thus proper appraisal of bioenergetic effects of mK_ATP_-channel opening using diazoxide as pharmacological tool requires the study of the direct effects of this drug on potassium transport in mitochondria.

A common methodical approach to the activation of mK_ATP_-channel by mK_ATP_-channel openers is based on preliminary blockage of the channel by Mg∙ATP and consequent activation, which for diazoxide lies in micromolar concentration area (~ 30 μM). Thus, functional effects of diazoxide as mK_ATP_-channel opener generally were assessed in the presence of Mg·ATP [13, 17]. The main limitation of this approach is that it interferes with the study of the effects of mK_ATP_-channel opening on potassium cycle because of the blockage of K^+^/H^+^-exchanger by Mg^2+^ ions [13]. For this reason, proper understanding of bioenergetic effects of diazoxide as well as diazoxide properties as mK_ATP_-channel opener, require the estimation of direct effects of diazoxide on potassium transport and mitochondrial functions in native mitochondria, in the absence of Mg∙ATP. The aim of this work was to revise the effects of diazoxide on mK_ATP_-channel activity, potassium transport, potassium cycle and ROS production in native rat liver mitochondria, in the absence of Mg∙ATP.

## Results

### The effect of diazoxide on state 4 oxygen consumption in rat liver mitochondria

**Fig. 1:**
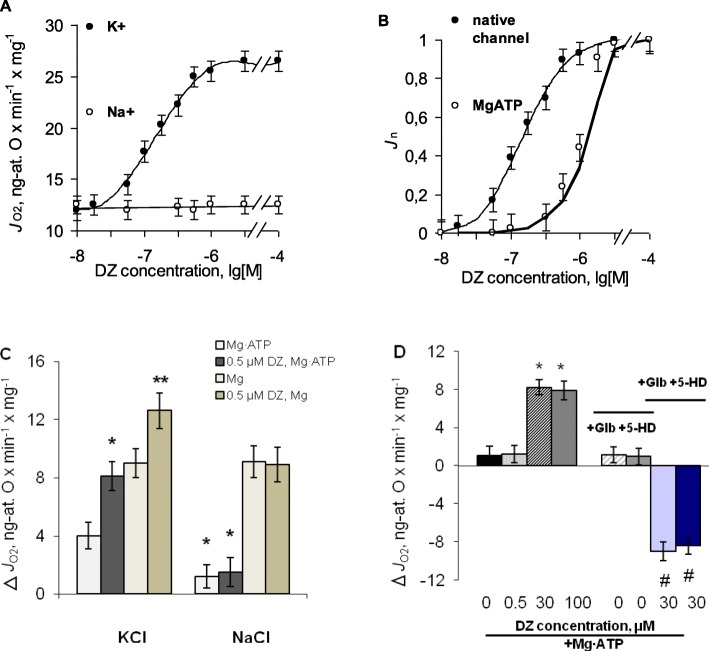
The effect of diazoxide (DZ) on state 4 respiration in rat liver mitochondria in the absence and the presence of MgATP. **a** – Respiration rates in K^+^- and Na^+^-based media against DZ concentration, lg [M]; **b** – normalized respiration rates in the absence (native mitochondria) and the presence of Mg∙ATP; **c** – respiration rate differences in K^+^- and Na^+^-based media sensitive to the addition of Mg∙ATP and Mg^2+^ in the absence and the presence of 0.5 μM DZ; **d** – respiration rate differences in the presence of Mg∙ATP sensitive to the addition of DZ, glibenclamide and 5-HD. Additions are shown in the legends. M ± m, *n* = 9. **c**, **d**: * - *P* < 0.05 vs. MgATP, 0 DZ; ** - *P* < 0.01 vs. Mg^2+^; # - P < 0.01 vs. MgATP, 30 μM DZ

Potassium transport is known to be coupled to the rate of state 4 respiration, so to test the effect of diazoxide on mitochondrial K^+^ transport, the effect of this drug on the respiration was studied. Based on polarographic records, in the absence of Mg∙ATP, diazoxide produced about twofold increase in the rate of glutamate-driven respiration (*J*_O2_) in native mitochondria, from 12.0 ± 1.0 to 26.0 ± 0.9 ng-at. O∙min^− 1^∙mg^− 1^ (Fig. 1a, black circles; Fig. 1 Suppl). The effect was not dependent on simultaneous or sequential additions of the respiratory substrate and diazoxide (Fig. 1b, Suppl). Also, as we observed earlier [18], no apparent depolarization of mitochondria by diazoxide was observed within the timeframes of the respiration assays. The effect of diazoxide on mitochondrial respiration was concentration dependent, specific for K^+^-based medium, and was not observed when K^+^ was isotonically replaced by Na^+^ (Fig. 1a, empty circles). In the absence of Mg∙ATP the activation of state 4 respiration occurred at submicromolar concentrations of the drug, ≤ 0.5 μM, whereas Mg∙ATP led to the shift of the activation curve to micromolar concentrations (Fig. 1b). Without Mg·ATP, at concentrations above ~ 0.5 μM, up to high micromolar level (~ 100 μM) no further stimulation of respiration by diazoxide was found (Fig. 1a, b).

To ascertain the effect of diazoxide on native mK_ATP_-channel activity, we determined its share in state 4 respiration from the respiration rate differences sensitive to the addition of Mg^2+^ and ATP (Fig. 1c, d, Suppl,) in the absence and the presence of 0.5 μM of diazoxide, which caused full stimulation effect of this drug on the respiration. Difference in the respiration rates sensitive to sequential additions of Mg^2+^ and ATP in K^+^-based medium without diazoxide have shown that the share of native mK_ATP_-channel in the respiration was 4.0 ± 1.0 ng-at. O∙min^− 1^∙mg^− 1^ (Fig. 1c) and this constituted about 30% of the total respiration rate. Under maximal respiration stimulation by diazoxide Mg·ATP-sensitive component of oxygen consumption rose to 7.9 ± 1.1 ng-at. O∙min^− 1^∙mg^− 1^ (Fig. 1c), which indicated twofold increase in ATP-sensitive K^+^ uptake. In Na^+^-based medium Mg^2+^ similarly suppressed respiration, consistent with the notion of the ability of Mg^2+^ ions to block Na^+^ uptake in mitochondria. However, in Na^+^-based medium none effects of diazoxide on Mg^2+^-sensitive and ATP-sensitive components of oxygen consumption were found (Fig. 1c), which proved specific modulation of MgATP-sensitive part of respiration related to mK_ATP_-channel activity.

In the presence of Mg∙ATP diazoxide failed to stimulate respiration in submicromolar concentration area (Fig. 1b, d), but full stimulation reached with micromolar concentrations of the drug [12] gave the same estimate of maximal mK_ATP_-channel activity (Fig. 1d). This was confirmed by the blockage of the channel activated by diazoxide in the presence of MgATP by its blockers, glibenclamide and 5-HD, which in the absence of diazoxide under the same conditions had no effect on respiration, but abolished the stimulation of the respiration reached with 30 μM diazoxide (Fig. 1d). Based on K/O stoichiometry of 10:1 for NADH-dependent substrates [19], the rate of ATP-sensitive potassium transport in rat liver mitochondria rose from ~ 40 to ~ 80 nmol K^+^∙min^− 1^∙mg^− 1^, which reflected maximal activity of the channel activated in the presence of MgATP. At high micromolar concentrations (up to 100 μM) diazoxide could not increase the share of mK_ATP_-channel in oxygen consumption anymore (Fig. 1d).

In spite of more than twofold increase in the rate of state 4 respiration by diazoxide, it was rather surprising that contribution of mK_ATP_-channel to oxygen consumption rested at the same level of ~ 30–33%, even at full respiration stimulation. As we observed, this was caused by simultaneous increment of ATP-insensitive component of respiration, found in standard K^+^-based medium from the difference in respiration rates sensitive to the addition of Mg^2+^ in the absence and the presence of diazoxide (Fig. 1c; Fig. 1c, d, Suppl). Since Mg^2+^ is known to block considerable part of Na^+^ and K^+^ transport, several types of K^+^ channels and K^+^/H^+^-exchange [13, 20], we supposed that the synchronized increase of both ATP-sensitive and ATP-insensitive constituents of state 4 respiration under the action of diazoxide (Fig. 1c) indicates the activation of ATP-insensitive potassium transport in parallel with mK_ATP_-channel opening. Thus, to verify this assumption more directly, we studied the effect of diazoxide on potassium transport using light absorbance technique.

### The effect of diazoxide on mitochondrial matrix volume

**Fig. 2:**
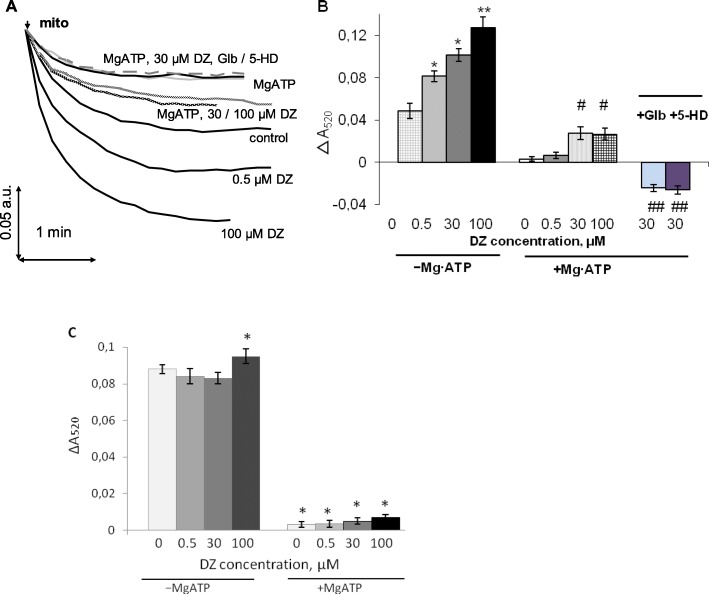
Absorbance assay of the effect of mK_ATP_-channel ligands on K^+^ transport. **a** – typical pattern in standard incubation medium with DZ added at 0, 0.5 and 100 μM (as indicated); Mg∙ATP (black line); Mg∙ATP and DZ at 30 and 100 μM (grey lines); Mg∙ATP, 30 μM DZ, glibenclamide (dotted line) or 5-HD (light grey); **b**, **c** − absorbance differences obtained in K^+^- (**b**) and Na^+^-(**c**) based media after the additions of DZ in the absence and the presence of Mg∙ATP (**b**, **c**); after the additions of glibenclamide and 5-HD in the presence of Mg∙ATP, 30 μM DZ (**b**). The values obtained in control in the presence of MgATP were subtracted. M ± m, *n* = 4; **b**, **c**: * - P < 0.05 vs. 0 DZ; ** - *P* < 0.01 vs. 30 μM DZ; # - *P* < 0.05 vs. MgATP, 0 DZ; ## - *P* < 0.01 vs. MgATP, 30 μM DZ

The effect of diazoxide on potassium transport, similar to oxygen consumption, was studied in the absence and the presence of Mg·ATP. In standard incubation medium without diazoxide considerable matrix swelling indicated potassium uptake by energized mitochondria (Fig. 2a). As we observed from absorbance assay, in K^+^-based medium mitochondrial swelling too was sensitive to submicromolar diazoxide concentrations (≤ 0.5 μM). However, unlike the respiration, it was progressively increased with the rise of diazoxide concentration up to high micromolar level (Fig. 2a) which indicated increase in total potassium uptake. Maximal swelling observed in this work with 100 μM DZ was a sort of a median between control and valinomycin (Fig. 2 Suppl). Thus to establish the share of mK_ATP_-channel in potassium transport we selectively activated mK_ATP_-channel by diazoxide in the absence and the presence of Mg∙ATP (Fig. 2b).

After the addition of Mg∙ATP to the medium, mitochondrial swelling was reduced to the minimum indicating essential block of potassium transport (Fig. 2a). Then, following Jaburek et al. [17], mK_ATP_-channel was fully activated by 30 μM of diazoxide (Fig. 2a). Difference in swelling amplitude sensitive to diazoxide stimulation in the presence of Mg∙ATP reflected maximal mK_ATP_-channel activity and its share in K^+^ transport (Fig. 2b). This was confirmed by the data showing the blockage of reactivated channel by glibenclamide and 5-HD (Figs. 2a, dotted lines; 2b). But while in the presence of Mg∙ATP diazoxide failed to activate mK_ATP_-channel anymore even at high micromolar concentrations (Figs. 2a and b), it exhibited potent activation of total potassium transport, starting from nanomolar concentrations without Mg∙ATP (Fig. 2b). When K^+^ was replaced by Na^+^, the activation of cation uptake was not observed either in the absence or the presence of MgATP, except weak stimulation found in the absence of MgATP at high diazoxide concentration, 100 μM (Fig. 2c). Similarly to K^+^ transport, Mg^2+^ blocked most part of the Na^+^ uptake, which agreed with the above oxygen consumption assay, but blocking effect was not dependent on diazoxide (Figs. 1, 2c). So, in support of our previous observations, absorbance assays strongly indicated the activation of both ATP-sensitive (mK_ATP_-channel) and ATP-insensitive potassium transport, highly susceptible to diazoxide activation in the absence of Mg∙ATP. However, unlike oxygen consumption assay, this caused the decrease of the partial share of ATP-sensitive potassium transport in total potassium uptake with the rise of diazoxide concentrations from nanomolar to high micromolar level (Fig. 2b). Thus, with the aim to ascertain the above observations independently, and quantify observed effects, we studied the effects of diazoxide on potassium transport using pH-sensitive probe BCECF.

### The effect of diazoxide on mK_ATP_-channel activity, potassium transport and potassium cycle in rat liver mitochondria

#### The effect of diazoxide on potassium uptake and mK_ATP_-channel activity

**Fig. 3:**
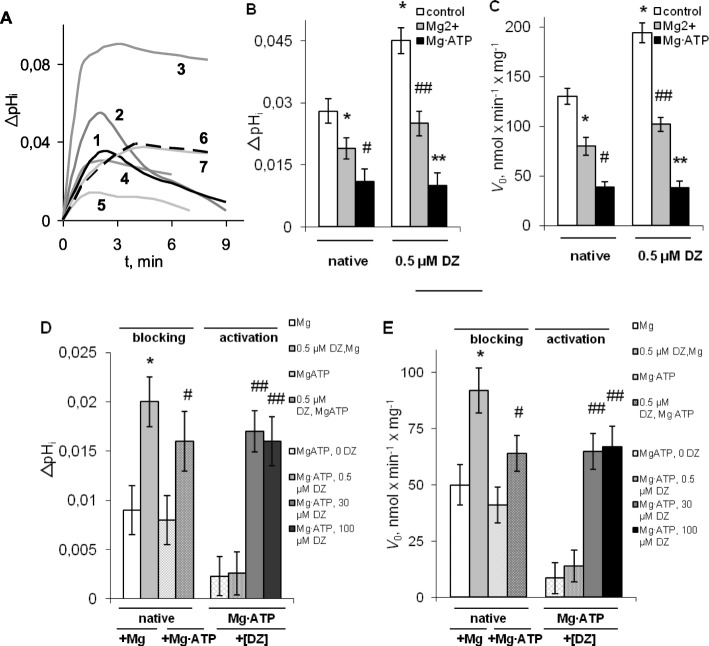
The effect of diazoxide on matrix pH and *V*_0_ of K^+^ uptake, in rat liver mitochondria. **a** – The typical changes in matrix pH (ΔpH_i_) in standard incubation medium with following additions: DZ at 0, 0.5, 100 μM (1–3); 0.5 μM DZ, 1 mM Mg^2+^ (4); Mg·ATP and DZ added at 0.5 (5), 30 (6) and 100 μM (7); **b**, **c** – the changes in ΔpH_i_ and *V*_0_ of K^+^ uptake after sequential blockage of K^+^ transport by Mg^2+^ and Mg·ATP in the absence (native mitochondria) and the presence of 0.5 μM DZ; **d**, **e** – the shares of ATP-sensitive and ATP-insensitive K^+^ transport in ΔpH_i_ and *V*_0_ of K^+^ uptake found from the blockage of K^+^ transport by Mg^2+^ and ATP in native mitochondria (−Mg·ATP) and the activation of mK_ATP_-channel in the presence of Mg·ATP (+Mg·ATP); DZ was added at 0.5 μM (−Mg·ATP); 0.5, 30 and 100 μM (+Mg·ATP). Additions are shown in the legend. M ± m, *n* = 6; **b**, **c**: * - P < 0.05 vs control (0 DZ); # - P < 0.05 vs. Mg2+; ## - P < 0.01 vs. 0.5 μM DZ; ** - P < 0.01 vs. Mg^2+^, 0.5 μM DZ; D, E: * - P < 0.05 vs. Mg^2+^ (native); # - P < 0.05 vs. MgATP (native) ## - P < 0.01 vs. MgATP, 0.5 μM DZ

The quantitative estimation of potassium transport was based on the notion that it is accompanied by the equivalent countertransport of protons, and simultaneous changes in matrix pH (pH_i_) [13, 21]. Representative curves of the change in matrix pH (ΔpH_i_), starting from the addition of mitochondria to the medium, are shown on the Figs. 3a and Fig. 3a Suppl. The changes in matrix pH and the initial rates of potassium uptake (*V*_0_) in native mitochondria after the successive blockage of proton transport by Mg^2+^ and ATP in the absence and the presence of diazoxide (Fig. 3b, c), were found with the purpose to obtain the estimates of ATP-insensitive (sensitive to Mg^2+^ only) and ATP-sensitive (sensitive to Mg·ATP) constituents of K^+^ transport (Fig. 3d, e). Also, the contribution of mK_ATP_-channel to potassium transport and ΔpH_i_ was estimated from the activation of the channel by diazoxide in the presence of Mg∙ATP (Fig. 3d, e).

**Fig. 4:**
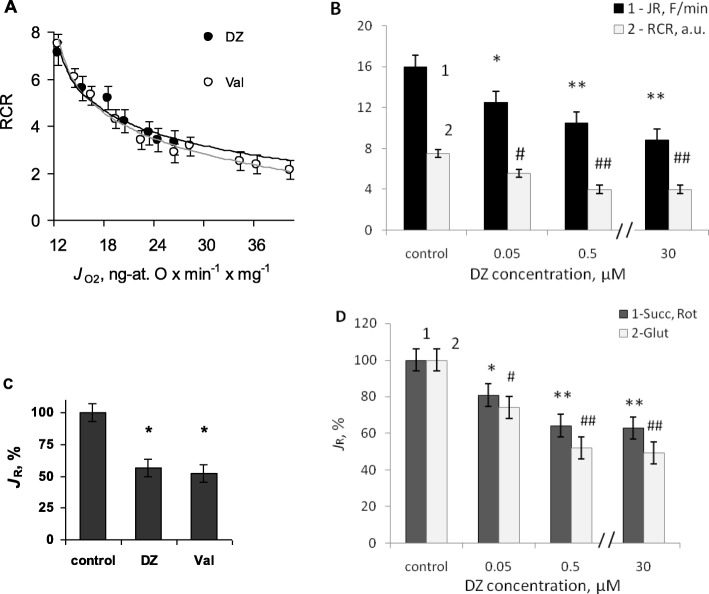
Bioenergetic effects of diazoxide: respiratory uncoupling (**a**) and suppression of ROS production (**b**-**d**). **a** – To assess uncoupling effect respiratory control ratio (RCR) was plotted against the rate of state 4 respiration (*J*_O2_) stimulated by diazoxide and valinomycin; **b** – the rates of oxidant formation found as the increments in DCF fluorescence (1) and mitochondrial uncoupling found as RCR (2) against DZ concentration, μM; **c** – the rates of ROS formation in tightly coupled mitochondria (RCR 7.5)), and under respiratory uncoupling by diazoxide and valinomycin (RCR 4.0); **d** – the rates of oxidant formation in mitochondria respiring on succinate (1) and glutamate (2). M ± m, n = 4; B-D: *, # - P < 0.05 vs.0 DZ; **, ## - P < 0.05 vs. 0.05 μM DZ

As it was found from the experiments, at the concentration of 0.5 μM shown to cause full activation of state 4 respiration in the absence of Mg^2+^ and ATP, diazoxide increased both matrix pH and *V*_0_ of proton transport related to K^+^ uptake (Fig. 3a, b, c and 2). In agreement with oxygen consumption data, Mg^2+^ ions blocked essential part of proton transport related to potassium uptake, which was suppressed further by Mg∙ATP, in parallel reducing ΔpH_i_ (Fig. 3a, b, c, 4, 5). From sequential blockage of potassium transport by Mg^2+^ and ATP (Fig. 3b, c), it was found that in the absence of Mg∙ATP native mK_ATP_-channel activity contributed about ~ 0.01 units change in pH_i_ and ~ 40 nmol∙min^− 1^∙mg^− 1^ to *V*_0_ of K^+^ uptake (Fig. 3d, e). Diazoxide at submicromolar concentration (0.5 μM) reliably increased *V*_0_ of both ATP-insensitive and ATP-sensitive potassium uptake in native mitochondria, with related changes in pH_i_ (Fig. 3d, e). Increase in *V*_0_ of Mg∙ATP-sensitive potassium transport from ~ 40 to 60 nmol∙min^− 1^∙mg^− 1^ (Fig. 3e) indicated the activation of native mK_ATP_-channel by sub-micromolar concentrations of this drug. Activity of native mK_ATP_-channel stimulated by low diazoxide concentrations without Mg·ATP well agreed with the estimate of the channel activity obtained with 30 μM diazoxide in the presence of Mg∙ATP, ~ 65 nmol∙min^− 1^∙mg^− 1^, with related changes in pH_i_ by ~ 0.015 units (Fig. 3d, e). Further increase of diazoxide concentration up to 100 μM failed to increase the channel activity anymore (Fig. 3a, 6–7; 3d, e), which was similar to oxygen consumption and absorbance assays.

Observed increase in state 4 oxygen consumption (Fig. 1 a) was in line with the notion of the activation of potassium cycle [13, 14]. To ascertain the ability of diazoxide to stimulate potassium cycling in sub-micromolar concentration area, we conducted the quantitative estimation of the effect of diazoxide on K^+^/H^+^-exchange in liver mitochondria.

#### The effect of diazoxide on mitochondrial K^+^/H^+^-exchange

In agreement with the published data [22] the elevation of matrix pH, caused by potassium uptake from standard incubation medium, typically was followed by matrix acidification (Fig. 3a, 1-3, Fig. 3a Suppl) and matrix contraction (Fig. 3b Suppl) which reflected the activity of K^+^/H^+^-exchanger. Proton transport was completely blocked by specific inhibitor of K^+^/H^+^-exchanger quinine (Fig. 3a Suppl). Both H^+^ influx and matrix contraction were activated by diazoxide (Figs. 3a;, 3a, b Suppl), which proved the activation of K^+^/H^+^-exchange and was confirmed further by the direct observation of K^+^ transport with the probe PBFI^1^ (Fig. 3c, d, Suppl).

Uptake and efflux of K^+^ were accompanied by the increase and the decrease in BCECF and PBFI fluorescence. As it was observed, DZ accelerated both phases of K^+^ cycle (Fig. 3a; Fig. 3c, d, Suppl).

Quinine blocked K^+^/H^+^-exchange independent on the presence of diazoxide (Fig. 3a Suppl), which was in line with the notion that K^+^/H^+^-exchange was the only pathway of K^+^ efflux from energized mitochondria under the conditions of our experiment. In the presence of quinine no changes in BCECF fluorescence after K^+^ uptake was observed (Fig. 3a Suppl), which allowed quantitative estimation of K^+^/H^+^-exchanger activity directly from proton transport data [24]. In support of our earlier results based on absorbance measurements showing mitochondrial contraction under the same conditions ([18] and Fig. 3b Suppl), the addition of diazoxide at ≤0.5 μM in the absence of Mg∙ATP resulted in the activation of K^+^/H^+^-exchange, as showed observed changes in kinetics of proton transport (Fig. 3a, 1, 2). Initial rate of quinine-sensitive potassium efflux was reliably increased by 0.5 μM of diazoxide from 23 nmol∙min^− 1^∙mg^− 1^ in control to maximal value of 34 nmol∙min^− 1^∙mg^− 1^. Thus activation of both phases of potassium cycle (Fig. 3c, d, Suppl) well agreed with maximal stimulation of state 4 respiration by diazoxide on the same sub-micromolar concentration scale. With the rise of diazoxide concentration above ~ 0.5 μM no further activation of K^+^/H^+^-exchange was observed [18], and even suppression of K^+^/H^+^-exchange by the high micromolar concentrations of the drug was observed (Fig. 3a, 3).

Mg^2+^ and Mg∙ATP dramatically suppressed the activity of quinine-sensitive K^+^/H^+^-exchange (Fig. 3a, 4–7; Fig. 3a, Suppl.), consistent with the notion of the blocking of K^+^/H^+^-exchanger by Mg^2+^ ions [13]. This could explain the absence of any apparent effect of diazoxide on K^+^/H^+^-exchange in the presence of Mg^2+^ and Mg∙ATP. Thus, the blockage of K^+^ uptake by successive additions of Mg^2+^ and Mg∙ATP, together with the inhibition of K^+^/H^+^-exchange by Mg^2+^ eventually resulted in a strong suppression of potassium cycle.

#### Non-specific effect of diazoxide on potassium transport

Similar to absorbance assays (Fig. 2a, b) gradual elevation of pH_i_ by increasing concentrations of diazoxide (from 0.5 to 100 μM) in the absence of Mg∙ATP indicated gradual increase of potassium uptake into matrix (Fig. 3a, 1-3). The sequential blockage of K^+^ transport by Mg^2+^ and Mg∙ATP allowed us observe that in native mitochondria diazoxide reliably activated a transport constituent sensitive to the blockage by Mg^2+^, which could be identified as ATP-insensitive K^+^ uptake (Fig. 3d, e).

Unlike mK_ATP_-channel, ATP-insensitive potassium transport was not restored by diazoxide in the presence of Mg^2+^ or Mg∙ATP. In fact, in the presence of Mg∙ATP diazoxide selectively restored only mK_ATP_-channel activity (Figs. 2b, 3d, e), which was proven by the complete blockage of the activated channel by glibenclamide and 5-HD (Figs. 1d, 2b). Since in the presence of Mg∙ATP increase in diazoxide concentration starting from ~ 30 μM up to high micromolar level had no more effect on mK_ATP_-channel activity (Fig. 2b, 3d, e), we came to the conclusion that the elevation of total potassium uptake in native mitochondria under high diazoxide concentrations, observed by absorbance (Fig. 2a, b) and BCECF fluorescence measurements (Fig. 3a, 1-3), occurred at the cost of ATP-insensitive potassium uptake. Proportion of ATP-insensitive to ATP-sensitive potassium transport (blocked respectively by Mg^2+^ and Mg·ATP) increased with the rise in diazoxide concentration, and starting from ~ 1:1 in submicromolar concentration area (Fig. 3d, e), reached ~ 3:1 at 100 μM of the drug (Fig. 3a, 3 vs. 3d, MgATP, 100 μM diazoxide). So, obtained results unambiguously indicated the ability of diazoxide to activate an ATP-insensitive potassium transport, which under experimental conditions was irreversibly blocked by Mg^2+^, and consequently, by Mg∙ATP.

### Bioenergetic effects of diazoxide in rat liver mitochondria

#### Uncoupling of mitochondrial respiration by diazoxide on sub-micromolar scale

As we have shown, on sub-micromolar concentration level diazoxide was effective in the activation of potassium cycle due to the enhancement of total K^+^ uptake and the activation of K^+^/H^+^ exchange. Potassium cycling is energy-dissipating process, thus to assess uncoupling effect of potassium cycle stimulated by diazoxide, the respiratory control ratio (RCR) was found at concentrations efficient in stimulation of state 4 respiration. In Fig. 4a obtained RCR values were plotted against the rate of state 4 respiration stimulated by increasing concentrations of diazoxide. RCR decrease with the increase in state 4 oxygen consumption indicated mitochondrial uncoupling (Fig. 4a, black circles). With the aim to ascertain that uncoupling by diazoxide resulted from the activation of potassium transport, we compared the effect of diazoxide to the effect of K^+^-ionophore, valinomycin. Based on RCR ratios, the same respiration stimulation by diazoxide and valinomycin resulted in similar mitochondrial uncoupling (Fig. 4a, white circles). This allowed the conclusion that uncoupling of the respiratory chain by diazoxide was caused by the stimulation of potassium cycle resulting from the activation of potassium uptake in mitochondria, without any non-specific effect.

Despite the lack of diazoxide selectivity as mK_ATP_-channel opener in the absence of Mg∙ATP, the estimation of the share of mK_ATP_-channel in state 4 oxygen consumption enabled us to assess partial contribution of the channel to mitochondrial uncoupling. Thus, from Fig. 4a, and the share of mK_ATP_-channel in state 4 respiration found earlier (~ 8 ng-at. O∙min^− 1^∙mg^− 1^), we obtained that maximal mK_ATP_-channel activity stimulated by diazoxide should reduce RCR in tightly coupled mitochondria by ~ 3.5 units, i.e. from ~ 7.5 to ~ 4.0, which was consistent with the notion of mild uncoupling of the respiratory chain [13].

#### mK_ATP_-channel opening by diazoxide on sub-micromolar level reduces ROS formation in liver mitochondria

To find the effect of diazoxide on ROS production in liver mitochondria, DCF fluorescence was monitored under steady-state conditions related to state 4 respiration. Quasi linear increase of fluorescence under these conditions was observed within ~ 5 min since the addition of mitochondria (Fig. 4a, b, Suppl). In non-respiring mitochondria DCF gave very low fluorescence signal, which apparently increased after the addition of the respiratory substrates (Fig. 4 a, b, Suppl), thus DCF fluorescence under experimental conditions largely reflected ROS formation by the respiratory chain. The increment of DCF fluorescence with time (*J*_R_) was plotted against diazoxide concentration (Fig. 4b).

In sub-micromolar concentrations, capable of activation of ATP-sensitive K^+^ transport without apparent mitochondrial depolarization, diazoxide in the absence of Mg·ATP reliably reduced the rate of DCF oxidation as compared to control (tightly coupled mitochondria, RCR 7.5 (Fig. 4b; Fig. 4 Suppl), which indicated the suppression of ROS production. Inhibition of the oxidants formation in the respiratory chain well correlated with the uncoupling effect of diazoxide on mitochondrial respiration (Fig. 4b). Considering that respiratory uncoupling was caused by the activation of potassium transport (Fig. 4a), we examined whether mitochondrial ROS production was similarly affected by valinomycin. As we observed, the same uncoupling by valinomycin (at RCR ratio of 4.0 units) similarly suppressed ROS production (Fig. 4 c). To ascertain our conclusion about the reduction of ROS production by diazoxide using glutamate as the respiratory substrate, we conducted the same study with succinate adding rotenone to abolish the reverse electron transport. Results obtained on the succinate-driven respiration confirmed the inhibition of ROS formation in parallel with the activation of K^+^ cycle (Fig. 4d). Reduction of ROS formation by diazoxide was as well observed after the addition of antimycin A to block electron transport at Complex III (the conditions known to promote ROS production in mitochondria (Fig. 4b Suppl)).

In the literature, ability of diazoxide to interfere with DCF fluorescence and increase its intensity independent of mK_ATP_-channel opening was shown [25]. However, in our work, on the contrary, DCF fluorescence was decreased by diazoxide. Besides, referenced work reported the effects of high micromolar concentrations of the drug; while in our work low sub-micromolar concentrations of diazoxide were used. As we have shown, DCF fluorescence was reliably reduced by as low as 0.05–0.5 μM of diazoxide, which was in line with the activation of K^+^ cycle (Fig. 4a, b). Close effect obtained with valinomycin under similar uncoupling conditions, independent of diazoxide (Fig. 4c) confirmed the conclusion that the decrease in DCF fluorescence can be explained by the reduction of ROS formation in mitochondria. Thus, we came to the conclusion that ROS production in native liver mitochondria in the absence of Mg·ATP was highly sensitive to low sub-micromolar diazoxide concentrations because of mK_ATP_-channel opening, activation of potassium cycling and respiratory uncoupling within the same concentration range.

## Discussion

### The effect of diazoxide on mK_ATP_-channel activity

To study the effect of diazoxide on K^+^ transport, two sets of experiments were conducted: in the absence and the presence of Mg·ATP. To ascertain specific activation of mK_ATP_-channel by submicromolar diazoxide concentrations (≤0.5 μM), potassium transport was sequentially blocked by Mg^2+^ and ATP. With the aim to establish the share of mK_ATP_-channel in total K^+^ uptake, ATP-sensitive K^+^ transport was activated by micromolar diazoxide concentrations in the presence of Mg∙ATP in agreement with [17] and then blocked by glibenclamide and 5-HD to prove mK_ATP_-channel opening. Without MgATP, diazoxide exhibited potent activation of K^+^ uptake. While in the presence of Mg·ATP mK_ATP_-channel was routinely activated by micromolar diazoxide concentrations (consistent with literary data), stepwise activation of K^+^ uptake by increasing concentrations of the drug in the absence of Mg∙ATP revealed high sensitivity of mK_ATP_-channel to this opener, with full activation at ≤0.5 μM. Additionally, parallel activation of ATP-insensitive potassium uptake was found that increased with the rise of diazoxide concentration up to 100 μM.

Worth mention, that main limitation of our study was the use of indirect methods to assess mK_ATP_-channel activity and inability of molecular identification of ATP-sensitive K^+^ transport sensitive to diazoxide in the absence of MgATP. However, an estimate of native mK_ATP_-channel activity (~ 40 nmol K^+^·min^− 1^·mg^− 1^) using respiration and proton transport assays well agreed with the assessment of mK_ATP_-channel activity with K^+^-selective electrode (45.0 ± 5.0 nmol K^+^·min^− 1^·mg^− 1^) by monitoring ATP-sensitive potassium efflux from deenergized mitochondria [26]. Also, sequential blockage of K^+^ transport by Mg^2+^ and ATP proved the activation of native mK_ATP_-channel by diazoxide. In the presence of MgATP, which blocked mK_ATP_-channel, no additional blocking of K^+^ transport by mK_ATP_-channel blockers glibenclamide and 5-HD was observed (Fig. 1d). This proves that ATP-sensitive K^+^ transport, activated by diazoxide and blocked by MgATP, can be ascribed to native mK_ATP_-channel activity in rat liver mitochondria.

Mg∙ATP was generally thought to be indispensable for mK_ATP_-channel activation by potassium channels openers, but in support of our findings, literary data as well showed susceptibility of mK_ATP_-channel to the activation by diazoxide and the blockage by glibenclamide and 5-HD in the absence of Mg·ATP [27, 28]. However, unlike the works referred to above, which used high micromolar diazoxide concentrations, we proved full stimulation of ATP-sensitive K^+^ transport on sub-micromolar scale. The sensitivity of native mK_ATP_-channel of rat liver mitochondria to diazoxide was close to literary data on isolated reconstituted mK_ATP_-channel with apparent activation constant *K*_a_ ~ 350 nM [12]. In our work, activation of the channel in the presence of Mg·ATP, even at high micromolar diazoxide concentrations (100 μM), could not exceed activity reached with native channel at submicromolar concentrations of the drug in the absence of Mg·ATP (Fig. 2a, b; Fig. 3d, e). Thus our experiments allow us hypothesize that mK_ATP_-channel might comprise the site(s) accessible to pharmacologic modulators and responsible for the channel activation in the absence of Mg·ATP.

### Side effect of diazoxide on potassium uptake in rat liver mitochondria

In the literature several side effects of diazoxide were reported, such as protonophoric uncoupling [15], respiratory inhibition [16], interference with flavoprotein oxidation [29] and DCF fluorescence [25]. The lack of diazoxide selectivity as mK_ATP_-channel opener found in our work, which was shown in parallel activation of ATP-insensitive potassium transport, was novel not described earlier side effect of this drug. ATP-insensitive potassium transport rose gradually with the increase of diazoxide concentration up to high micromolar level. Accordingly, total potassium uptake under the action of diazoxide increased because of the increase in ATP-insensitive component (Figs. 2a, 3a). This resulted in the reduction of the ratio of ATP-sensitive to ATP-insensitive potassium uptake with the rise of diazoxide concentration. Unlike ATP-sensitive potassium transport, ATP-insensitive one could not be restored by diazoxide and its blockage by Mg^2+^ and Mg·ATP, was irreversible under experimental conditions.

For the most part unspecific actions of diazoxide reported in the literature [10] were observed at high micromolar concentrations (≥50–100 μM), and the side effects of diazoxide were independent of its action on potassium transport. Unlike the published data, side effect of diazoxide found in our work was the activation of ATP-insensitive potassium transport, starting from low sub-micromolar concentrations in the absence of Mg^2+^ and ATP. The nature of this transport remains yet to be established. Thus, the ability of diazoxide to activate other than K_ATP_ channels, BK_Ca_ and Kv channels, of plasma membrane of smooth muscle cells was reported [30]. But leaving aside several types of potassium channels already described in mitochondria (e.g. in the review [11]), even in the case of mK_ATP_ channel, uncertainty still exists about molecular mechanism of its response to diazoxide [31, 32].

Generally, it is known that K_ATP_-channels openers bind to the receptor SUR subunits of the channel, which possesses MgATPase activity. So, the presence of MgATP is considered to be indispensable for K_ATP_-channel opening [12]. Meanwhile, none of the recent hypotheses about molecular nature of K^+^ conductant subunit of mK_ATP_-channel could satisfactory explain the mechanism of mK_ATP_-channel’s response to pharmacological openers, such as diazoxide [31, 32]. As it was reported in earlier studies, in the heart, Kir6.2 was dispensable for the K^+^ conductance stimulated by diazoxide [31]. Also, none of ROMK isoforms (Kir1.1, Kir3.1, and Kir3.4) proposed as K^+^ conductant subunit of mK_ATP_-channel [33, 34] was responsible for diazoxide-induced swelling of cardiac mitochondria [32]. These studies indicated that K^+^ conductant subunit of mK_ATP_-channel didn’t belong to either Kir6.x, or Kir1.x channels, but molecular nature of the K^+^ pore of mK_ATP_-channel remained elusive.

However, quite recently, molecular composition of mK_ATP_-channel was disclosed, based on combined proteomics, biophysical and biochemical studies [35]. This work principally confirmed the knowledge on molecular architecture of mK_ATP_-channel as an octameric multiprotein complex composed of four K^+^ conductant and four receptor subunits, named MITOK and MITOSUR respectively. Also, it was confirmed that K^+^ conductant subunit of mK_ATP_-channel performs a number of vital functions, such as volume regulation, maintenance of mitochondrial membrane potential, regulation of ATP synthesis and Ca^2+^ transport [35]. Genetic deletion of MITOK caused instability of ΔΨm, suppression of phosphorylation, and loss of cardioprotective effect of diazoxide [35].

Meanwhile, novel discovery of mK_ATP_-channel put novel questions about the functions of Kir6.x and Kir1.x channels, which expression was found in cardiac, brain, and other tissues [33]. Also, still much controversy remains about the properties of mK_ATP_-channel, their unexplained diversity in different preparations (mitoplasts, proteoliposomes, isolated mitochondria), and uncertainty about the direct and off-target effects of pharmacological modulators of mK_ATP_-channel. The disclosure of molecular nature of mK_ATP_-channel will help in filling numerous gaps in the understanding of the properties of mK_ATP_-channels, their physiological functions, and molecular mechanisms of their interactions with pharmacological modulators.

Thus, to explain observed effects of diazoxide on ATP-sensitive K^+^ transport, we cannot rule out some particular mode of the activation of mK_ATP_-channel in the absence of Mg·ATP. Also, the blockage of ATP-insensitive inward potassium transport by Mg^2+^ alone, in the absence of ATP implies the involvement of some other types of potassium channels in the activation of K^+^ transport by diazoxide.

The inability of diazoxide to stimulate state 4 respiration at concentrations above ~ 0.5 μM (Fig. 1a), differed from our transport assays, which showed gradual increase of K^+^ uptake with the increase of diazoxide concentration. Based on proton transport study, we observed that lack in respiration stimulation with the increase in diazoxide concentration above a certain threshold well coincided with the failure of this drug in stimulation of K^+^/H^+^-exchange at concentrations above ~ 0.5 μM. Moreover, a suppression of K^+^/H^+^-exchange was observed with the increase of diazoxide concentration to high micromolar level (Fig. 3a, 3). However, on sub-micromolar scale, “mild” uncoupling of respiratory chain by diazoxide was shown to result directly from the activation of potassium transport, which was confirmed by close proximity of the effects of diazoxide and K^+^ ionophore valinomycin (Fig. 4a).

### Suppression of ROS production by diazoxide in liver mitochondria

The modulation of ROS production by diazoxide and its cytoprotective effects was shown in multiple studies. However, these works used high diazoxide concentrations in the presence of MgATP [22]. The aim of this work was to examine the effects of low diazoxide concentrations, capable of full stimulation of mK_ATP_-channel activity without MgATP, on mitochondrial ROS production. As we have shown in this work, rat liver mitochondria exhibited high sensitivity of ROS production to diazoxide at sub-micromolar concentration level explained by high sensitivity of potassium transport to this drug.

It worth mention, that published data showed much controversy regarding the effect of diazoxide and other mK_ATP_-channel openers on ROS production in different tissues. While many authors reported the increase of ROS production caused by mK_ATP_-channels opening [1, 2, 22, 36], other studies showed the decrease of free radical formation caused either by diazoxide, or mK_ATP_-channel opening under physiological and pathophysiological conditions in the heart [3, 7], brain [3] and liver [6]. But while in the brain the decrease of ROS production can be explained by appreciable mitochondrial depolarization due to potassium transport [37], in heart and liver mK_ATP_-channel opening was unable to cause depolarization sensed by commonly used techniques such as potentiometry or fluorescent probes [13]. Thus it was proposed that the reduction of ROS production caused by mK_ATP_-channels openers could be explained by mild mitochondrial uncoupling and minor depolarizations (~ 2–5 mV) caused by ATP-sensitive K^+^ uptake [6, 25]. While we did not observe any apparent effect of diazoxide on ΔΨ_m_, our data in line with this assumption, showed a good correlation between mitochondrial uncoupling and the decrease of ROS formation in the course of forward electron transport driven either by glutamate or succinate (Fig. 4b-d). Thus minor fluctuations in ΔΨ_m_ caused by the uncoupling of the respiratory chain remain a plausible explanation for the observed decrease of ROS production by sub-micromolar concentrations of diazoxide in liver mitochondria under several conditions examined in this work (Fig. 4 Suppl). Worth notion, that the mechanisms controlling ROS production in mitochondria exhibited large differences dependent on the cell types [38]. So, the mechanisms in which diazoxide affects ROS production as well can show cell-specificity. However, detailed study is required to answer this issue.

Similar to what was observed by Garlid’s group [22], in our work matrix pH was increased by diazoxide (Fig. 3a, 1, 2), which is known to promote ROS production [39]. But unlike Garlid’s works, in our study diazoxide suppressed ROS production within the timeframes coincident with the elevation of matrix pH (Fig. 3a, 1, 2; Fig. 4a, b Suppl). In our case, this difference can be explained by strong activation of K^+^/H^+^-exchange, and mitochondrial uncoupling caused by the activation of K^+^ cycle, which was suppressed by MgATP in the works of Garlid’s group. As we observed, in the presence of MgATP diazoxide in micromolar concentrations increased K^+^ uptake by the opening of mK_ATP_-channel, but failed to restore K^+^/H^+^-exchange in mitochondria (Fig. 3a, 6, 7). Matrix alcalinization caused by the blocking of K^+^/H^+^-exchange in the presence of MgATP could promote the elevation of ROS production by mK_ATP_-channel openers observed in Garlid’s works [1, 22].

## Conclusions

In this work using indirect methods to study K^+^ transport and mK_ATP_-channel actiuvity, we disclosed novel aspects of native mK_ATP_-channel opening by diazoxide, and the ability of this drug to increase ATP-insensitive potassium transport in mitochondria in the absence of Mg∙ATP. Based on the experiments we established the following: 1) high sensitivity of native mK_ATP_-channel to the activation by diazoxide with maximal effect at ≤0.5 μM, which strongly affects mitochondrial bioenergetics on submicromolar scale; 2) strong activation of ATP-insensitive potassium transport, which was novel, not described earlier side effect of the drug; 3) high sensitivity of state 4 oxygen consumption to diazoxide at ≤0.5 μM caused by the activation of potassium cycle in the absence of MgATP and 4) suppression of ROS production caused by the activation of K^+^ cycle and mitochondrial uncoupling within the same sub-micromolar concentration range. Based on the experiments, we hypothesized the presence of high affinity sites for diazoxide binding. Their possible screening by MgATP shifts mK_ATP_-channel affinity to diazoxide from sub-micromolar to micromolar concentration scale.

Mitochondrial potassium channels, together with uncoupling proteins represent potent uncoupling machinery [14], which protects mitochondria from ROS overproduction and Ca^2+^ overload. The results obtained in our work make ground for using diazoxide as an effective pharmacological tool for the modulation of mitochondrial bioenergetics and ROS production. High sensitivity of mK_ATP_-channel to diazoxide in the absence of ATP observed in this study allows us hypothesize that under pathophysiological states and conditions marked by ATP deficiency (such as hypoxia and ischemia) affinity of mK_ATP_-channel to this drug can increase several times, which might contribute to the high effectiveness of diazoxide in cardio- and neuroprotection. This may be of relevance for health care practice, based on the report of successful application of sub-maximal doses of diazoxide for neuroprotection published in the literature [40].

Molecular mechanism of mK_ATP_-channel activation by diazoxide and other mK_ATP_-channel openers was not yet established. Our experiments reveal novel aspects of mK_ATP_-channel interaction with diazoxide which would help bring new insight into understanding of mK_ATP_-channel properties.

## Methods

For the purpose to study the effect of mK_ATP_-channel opener diazoxide on native mK_ATP_-channel activity, potassium transport, and potassium cycle, the study was conducted in parallel, in the absence and in the presence of MgATP. To assess the mK_ATP_-channel activity, the channel was conventionally blocked by MgATP, opened by high micromolar concentrations of diazoxide, and repeatedly blocked by mK_ATP_-channel blockers glibenclamide and 5-HD [17]. To assess the effect of diazoxide on mK_ATP_-channel activity without MgATP, potassium transport was sequentially blocked by Mg^2+^ and ATP. ATP-sensitive part of K^+^ transport was assumed to reflect mK_ATP_-channel activity. The rate of ATP-sensitive K^+^ transport found without MgATP was compared to mK_ATP_-channel activity obtained in the presence of MgATP.

### Mitochondrial preparations

The work has been conducted in accordance with “Guide for the Care and Use of Laboratory Animals” 8th ed. Washington, DC: National Research Council of the National Academies: The National Academic Press, 2011. All procedures involving animals were approved by the Ethics Commission on Animal Experiments of A.A. Bogomoletz Institute of Physiology, NAS of Ukraine. Adult Wistar-Kyoto female rats with 180–200 g mean body weight, kept in plexiglass cages at 12/12-h light/dark cycle (22 °C), and fed on standard diet with free access to water, were obtained from Veterinary department of AA Bogomoletz Institute of Physiology.

Prior to sacrifice, the animals were lightly anesthetized with ether inhalation and decapitated. Single animal was used in each experiment. Liver was excised and washed by cold 0.9% KCl solution (4 °C), minced and homogenized in 1:5 volume of the isolation medium: 250 mM sucrose, 1 mM EDTA, 20 mM Tris-HCl buffer, 4 °C (pH 7.2). Mitochondria were isolated by centrifugation at 700 x g for 7 min (4 °C). After the pellet was discarded, supernatant was centrifuged again at 11000 x *g* for 15 min (4 °C). Final pellet was resuspended in a small volume of isolation medium without EDTA and stored on ice. The protein content was determined by the Lowry method.

### The study of oxygen consumption

Oxygen consumption was studied polarographically in 1 cm^3^ closed termostated cell at 26 °C with platinum electrode at constant stirring in standard incubation medium: 120 mM KCl, 0.5 mM EDTA, 5 mM sodium glutamate, 1 mM KH_2_PO_4_, and pH was adjusted to 7.4 by KOH. When necessary, K^+^ was isotonically replaced by Na^+^; in the presence of Mg^2+^ EDTA was replaced by EGTA. mK_АТР_-channel activity was assessed as ATP-sensitive component of state 4 respiration, based on its specific blockage by Mg∙ATP, reactivation by diazoxide and repeated blockage with glibenclamide and 5-hydroxydecanoate, 5-HD [17]. It was found as an absolute value of respiration rate differences sensitive to the addition of ATP in the presence of Mg^2+^; diazoxide in the presence of Mg∙ATP, or glibenclamide and 5-HD in the presence of Mg∙ATP and 30 μM diazoxide. When required, other additions to standard incubation medium were as follows: MgCl_2_ (1 mM), ATP (0.3 mM), glibenclamide (10 μM), 5-HD (100 μM), oligomycin (1 μg/mg protein), ADP (200 μM). Diazoxide was added at concentrations required. Cytochrome *c* was added to standard incubation medium at 10 μM. Respiratory control ratio (RCR) was found as the ratio of state 3 to state 4 respiration rates. Final protein concentration was 1.5–2.0 mg/ml.

### The light absorbance assay of potassium transport

The effect of diazoxide on potassium transport was assessed spectrophotometrically by monitoring the decxrease in the absorbance of mitochondrial suspensions caused by potassium uptake and matrix swelling [17]. Absorbance was monitored at 520 nm in 1 cm^3^ cell in standard incubation medium starting from the addition of mitochondria at 1.0 mg/ml. Other additions were as described above. The total absorbance change reached since the addition of mitochondria to the incubation medium was ascribed to matrix swelling. In order to assess the effects of mK_ATP_-channel ligands on the channel activity, absorbance change differences (ΔA) were found as the differences in swelling amplitude sensitive to diazoxide and mK_ATP_-channel blockers, glibenclamide and 5-HD, in the absence and the presence of Mg·ATP.

### The study of proton transport

Proton transport and the change in matrix pH (ΔpH_i_) were assessed with pH-sensitive fluorescent probe 2′,7′-bis-(2-carboxyethyl)-5(6)-carboxyfluorescein acetoxymethyl ester (BCECF-AM) according to Brierley and Jung [24]. Briefly, mitochondria were preloaded with BCECF-AM (final concentration 10 μM) and incubated 10 min at 37 °C, then washed from the excess dye and stored on ice (4 °C). After addition of aliquots of the suspension (0.3 mg/ml) to standard incubation medium, the change in BCECF fluorescence because of proton transport was recorded at the excitation/emission wavelengths 509/535 nm. Basal fluorescence (F_0_) caused by the addition of mitochondria to the incubation medium was subtracted. F_0_ was found by extrapolation of kinetic curves to zero time. For the quantitative estimation of the initial rates of proton transport (*V*_0_) aliquots of the suspension loaded by BCECf were titrated by HCl in standard incubation medium in the presence of 5∙10^− 6^ M rotenone and 10^− 6^ M CCCP without EDTA. The changes in matrix pH (ΔpH_i_) were determined in parallel under the same conditions with a glass microelectrode in 1 cm^3^ volume of the medium. Initial rates of proton transport (nmol H^+^∙min^− 1^∙mg^− 1^) and the values of ΔpH_i_ were determined from the calibration curves obtained in the absence and the presence of Mg^2+^ and Mg∙ATP. In case of Mg∙ATP free concentration of H^+^ ions was obtained from parallel titrations with glass microelectrode measuring pH in the absence and the presence of Mg∙ATP.

The rates of potassium transport were found from the rates of proton transport, based on the known stoichiometry of 1H^+^:1 K^+^. K^+^/H^+^ exchange was assessed directly by monitoring quinine-sensitive decrease in BCECF fluorescence reflecting proton influx to the matrix [24].

### The detection of ROS formation in mitochondria

To study the effect of diazoxide on ROS formation, widely used probe 2′,7′-dichlorofluorescein diacetate (DCFH_2_DA) was applied. This compound readily penetrates mitochondrial membranes following the concentration gradient and is deacetylated in the matrix to form membrane-impermeable non-fluorescent derivative H_2_DCF (2′,7′-dihydrodichlorofluorescein), which is oxidized by mitochondrial ROS with the formation of highly fluorescent end product 2′,7′-dichlorofluorescein, DCF [41]. Mitochondria in stock suspension (20 mg/ml) were loaded with 200 μM of DCFH_2_DA for 30 min at 37 °C in the dark, then washed of excess probe and stored on ice (4 °C). After addition of mitochondria at 1 mg/ml, the increase in DCF fluorescence reflecting ROS formation was monitored over 5 min time interval, based on polarographic monitoring of the rate of state 4 respiration, which is inhibited with time because of gradual release of cytochrome c from mitochondria. Under the conditions when steady state rate of respiration was established, the time courses of DCF fluorescence exhibited quasi linear increase in fluorescence intensity (Fig. 4 A, B Suppl). The rate of the increase in DCF fluorescence and the change in fluorescence intensity over the time of observation (3 min) was assumed to reflect ROS formation in mitochondria.

### Chemicals

All reagents were from Sigma-Aldrich, USA. Deionized water was used for solutions preparations.

### Statistical analysis

The data were expressed as mean ± S.D. of 4–6 independent experiments. Statistical analysis was performed based on one-way and two-way ANOVA statistics followed by Bonferroni multiple comparisons test. *P* < 0.05 was considered as statistically significant. For two-way ANOVA statistics computer program supplied by the site www.wessa.net [42] was used.

## Supplementary information

**Additional file 1: Figure S1.** Typical polarographic records showing the effect of diazoxide on the glutamate driven respiration of rat liver mitochondria. On the curves are shown the additions to standard incubation medium, the rates of respiration in ng-at. O·min^− 1^·mg^− 1^, and the sequence of additions. **Figure S2.** The effect of diazoxide and valinomycin on the absorbance of mitochondrial suspension: A - typical time courses of mitochondrial swelling in standard incubation medium (control, 1) and after the addition of valinomycin (2) and diazoxide (3); B – absorbance changes under the same conditions. The data are mean of 3 independent experiments (*n* = 3; M ± m; * - P < 0.05). **Figure S3.** A, B – typical traces showing the time courses of BCECF fluorescence and absorbance in the absence (control) and the presence of 0.5 μM of diazoxide. Other additions are shown on the legends; quinine was added at 0.5 mM, MgCl_2_ at 1 mM. C, D – the typical changes in PBFI fluorescence in the absence (1, 2) and the presence of DZ (3, 4); * - P < 0.05 (3, 4 vs. 1, 2). **Figure S4.** A, B: Typical time courses of DCF fluorescence in rat liver mitochondria in standard incubation medium with the additions described in the legends. C: the changes in DCF fluorescence over 4 min of incubation. The data are means of 4 independent experiments (*n* = 4; M ± m; * - P < 0.05 as compared to controls without DZ).

## Data Availability

The datasets used and/or analyzed during this study are available from the corresponding author on reasonable request.

## Abbreviations

mK_ATP_-channel: Mitochondrial ATP-sensitive K^+^ channel
DZ: Diazoxide
5-HD: 5-hydroxydecanoate
ROS: Reactive oxygen species
DCF: Dichlorofluorescein
BCECF: 2′,7′-bis-(2-carboxyethyl)-5(6)-carboxyfluorescein
PBFI: Potassium-binding benzofuran isophtalate
RCR: Respiratory control ratio

## Footnotes

1 For this purpose mitochondria were loaded with 20 μM PBFI-AM for 20 min at room temperature. PBFI fluorescence was monitored at 345/485 excitation/emission wavelengths [23]. The data of two independent assays are in Fig. 3c, d Suppl.

## References

[1] Garlid KD, Costa AD, Quinlan CL, Pierre SV, Dos Santos P (2009). Cardioprotective signaling to mitochondria. J Mol Cell Cardiol.

[2] Daiber A (1797). Redox signaling (cross-talk) from and to mitochondria involves mitochondrial pores and reactive oxygen species. Biochim Biophys Acta.

[3] Zhang F, Cui J, Lv B, Yu B (2015). Nicorandil protects mesenchymal stem cells against hypoxia and serum deprivation-induced apoptosis. Int J Mol Med.

[4] Lucas AM, Caldas FR, da Silva AP, Ventura MM, Leite IM, Filgueiras AB, Silva CG, Kowaltowski AJ, Facundo HT (2016). Diazoxide prevents reactive oxygen species and mitochondrial damage, leading to anti-hypertrophic effects. Chem Biol Interact.

[5] Rozova EV, Mankovskaya IN, Belosludtseva NV, Khmil NV, Mironova GD (2019). Uridine as a protector against hypoxia-induced lung injury. Sci Rep.

[6] Alberici LC, Oliveira HCF, Paim BA, Mantello CC, Augusto AC, Zecchin KG, Gurgueira SA, Kowaltowski AJ, Vercesi AE (2009). Mitochondrial ATP-sensitive K^+^ channels as redox signals to liver mitochondria in response to hypertriglyceridemia. Free Radic Biol Med.

[7] Liang W, Chen M, Zheng D, Li J, Song M, Zhang W, Feng J, Lan J (2017). The opening of ATP-sensitive K^+^ channels protects H9c2 cardiac cells against the high glucose-induced injury and inflammation by inhibiting the ROS-TLR4-necroptosis pathway. Cell Physiol Biochem.

[8] Storey NM, Stratton RC, Rainbow RD, Standen NB, Lodwick D (2013). Kir6.2 limits Ca (2+) overload and mitochondrial oscillations of ventricular myocytes in response to metabolic stress. Am J Physiol Heart Circ Physiol.

[9] Mironova GD, Khrenov MO, Talanov EY, Glushkova OV, Parfenyuk SB, Novoselova TV, Lunin SM, Belosludtseva NV, Novoselova EG, Lemasters JJ (2018). The role of mitochondrial KATP channel in anti-inflammatory effects of uridine in endotoxemic mice. Arch Biochem Biophys.

[10] Coetzee WA (2013). Multiplicity of effectors of the cardioprotective agent, diazoxide. Pharmacol Ther.

[11] Laskowski M, Augustynek B, Kulawiak B, Koprowski P, Bednarczyk P, Jarmuszkiewicz W, Szewczyk A (1857). What do we know about mitochondrial potassium channels?. Biochim Biophys Acta.

[12] Garlid KD, Paucek P, Yarov-Yarovoy V, Sun X, Schindler PA (1996). The mitochondrial K_ATP_ channel as a receptor for potassium channel openers. J Biol Chem.

[13] Garlid KD, Paucek P (1606). Mitochondrial potassium transport: the K^+^-cycle. Biochim Biophys Acta.

[14] Jarmuszkiewicz W, Szewczyk A (2019). Energy-dissipating hub in muscle mitochondria: potassium channels and uncoupling proteins. Arch Biochem Biophys.

[15] Kowaltowski AJ, Seetharaman S, Paucek P, Garlid KD (2001). Bioenergetic consequences of opening ATP-sensitive K^+^-channel of heart mitochondria. Am J Phys.

[16] Dröse S, Brandt U, Hanley PJ (2006). K^+^-independent actions of diazoxide question the role of inner membrane KATP channels in mitochondrial cytoprotective signaling. J Biol Chem.

[17] Jaburek M, Yarov-Yarovoy V, Paucek P, Garlid KD (1998). State-dependent inhibition of the mitochondrial K_ATP_ channel by glyburide and 5-hydroxydecanoate. J Biol Chem.

[18] Akopova OV, Nosar VI, Bouryi VA, Mankovska IN, Sagach VF (2010). Influence of ATP-dependent K^+^-channel opener on K^+^-cycle and oxygen consumption in rat liver mitochondria. Biochem Mosc.

[19] Beavis AD (1987). Upper and lower limits of the charge translocation stoichiometry of mitochondrial electron transport. J Biol Chem.

[20] Gelband CH, Ishikawa T, Post JM, Keef KD, Hume JR (1993). Intracellular divalent cations block smooth muscle K^+^ channels. Circ Res.

[21] Massari S, Azzone GF (1970). The mechanism of ion translocation in mitochondria. Coupling of K^+^ and H^+^ fluxes. Eur J Biochem.

[22] Andrukhiv A, Costa AD, West IC, Garlid KD (2006). Opening mitoK_ATP_ increases superoxide generation from complex I of the electron transport chain. Am J Phys.

[23] Bajgar R, Seetharaman S, Kowaltowski AJ, Garlid KD, Paucek P (2001). Identification and properties of a novel intracellular (mitochondrial) ATP-sensitive potassium channel in brain. J Biol Chem.

[24] Brierley GP, Jung DW (1990). Kinetic properties of the K^+^/H^+^ antiport of heart mitochondria. Biochemistry..

[25] Facundo HTF, Carreira RS, de Paula JG, Santos CCX, Ferranti R, Laurindo FRM, Kowaltowski AJ (2006). Ischemic preconditioning requires increases in reactive oxygen release independent of mitochondrial K^+^ channel activity. Free Radic Biol Med.

[26] Akopova O, Nosar V, Gavenauskas B, Bratus L, Kolchinskaya L, Mankovska I, Sagach V (2016). (2016) the effect of ATP-dependent potassium uptake on mitochondrial functions under acute hypoxia. J Bioenerg Biomembr.

[27] Riess ML, Camara AK, Heinen A, Eells JT, Henry MM, Stowe DF (2008). KATP channel openers have opposite effects on mitochondrial respiration under different energetic conditions. J Cardiovasc Pharmacol.

[28] Kopustinskiene DM, Liobikas J, Skemiene K, Malinauskas F, Toleikis A (2010). Direct effects of K_ATP_ channel openers pinacidil and diazoxide on oxidative phosphorylation of mitochondria *in situ*. Cell Physiol Biochem.

[29] Pasdois P, Beauvoit B, Tariosse L, Vinassa B, Bonoron-Adele S, Dos Santos P (2008). Effect of diazoxide on flavoprotein oxidation and reactive oxygen species generation during ischemia-reperfusion: a study on Langendorff perfused rat hearts using optic fibers. Am J Phys.

[30] Adebiyi A, McNally EM, Jaggar JH (2008). Sulfonylurea receptor-dependent and -independent pathways mediate vasodilation induced by ATP-sensitive K^+^ channel openers. Mol Pharmacol.

[31] Wojtovich AP, Urciuoli WR, Chatterjee S, Fisher AB, Nehrke K, Brookes PS (2013). Kir6.2 is not the mitochondrial KATP channel but is required for cardioprotection by ischemic preconditioning. Am J Phys.

[32] Henn MC, Janjua MB, Kanter EM, Makepeace CM, Schuessler RB, Nichols CG, Lawton JS (2015). Adenosine triphosphate-sensitive potassium channel Kir subunits implicated in cardioprotection by diazoxide. J Am Heart Assoc.

[33] Foster DB, Ho AS, Rucker J, Garlid AO, Chen L, Sidor A, Garlid KD, O'Rourke B (2012). Mitochondrial ROMK channel is a molecular component of mitoK (ATP). Circ Res.

[34] Laskowski M, Augustynek B, Bednarczyk P, Żochowska M, Kalisz J, O'Rourke B, Szewczyk A, Kulawiak B (2019). Single-Channel Properties of the ROMK-Pore-Forming Subunit of the Mitochondrial ATP-Sensitive Potassium Channel. Int J Mol Sci.

[35] Paggio A, Checchetto V, Campo A, Menabò R, Di Marco G, Di Lisa F, Szabo I, Rizzuto R, De Stefani D (2019). Identification of an ATP-sensitive potassium channel in mitochondria. Nature..

[36] Oldenburg O, Cohen MV, Yellon DM, Downey JM (2002). Mitochondrial K (ATP) channels: role in cardioprotection. Cardiovasc Res.

[37] Akopova OV, Kolchinskaya LI, Nosar VI, Bouryi VA, Mankovska IN, Sagach VF (2014). Effect of potential-dependent potassium uptake on production of reactive oxygen species in rat brain mitochondria. Biochemistry (Mosc).

[38] Tahara EB, Navarete FD, Kowaltowski AJ (2009). Tissue-, substrate-, and site-specific characteristics of mitochondrial reactive oxygen species generation. Free Radic Biol Med.

[39] Liu S-S (1999). Cooperation of a “reactive oxygen cycle” with the Q cycle and the proton cycle in the respiratory chain – superoxide generating and cycling mechanisms in mitochondria. J Bioenerg Biomembr.

[40] Salgado-Puga K, Rodríguez-Colorado J, Prado-Alcalá RA, Peña-Ortega F (2017). Subclinical doses of ATP-sensitive potassium channel modulators prevent alterations in memory and synaptic plasticity induced by amyloid-β. J Alzheimers Dis.

[41] Karlsson M, Kurz T, Brunk UT, Nilsson SE (2010). Frennesson CI (2010) what does the commonly used DCF test for oxidative stress really show?. Biochem J.

[42] Wessa P. Free statistics software, Office for research development and education, version 1.2.1. 2020; URL https://www.wessa.net/.

