# Supplementary material for: Diazoxide affects mitochondrial bioenergetics by the opening of mKATP channel on submicromolar scale
Source: BMC Mol Cell Biol. 2020 Apr 19;21:31. doi: 10.1186/s12860-020-00275-0 (PMC7168813; doi:10.1186/s12860-020-00275-0)
Supplement: Supplementary file 1 — Additional file 1: Figure S1. Typical polarographic records showing the effect of diazoxide on the glutamate driven respiration of rat liver mitochondria. On the curves are shown the additions to standard incubation medium, the rates of respiration in ng-at. O·min− 1·mg− 1, and the sequence of additions. Figure S2. The effect of diazoxide and valinomycin on the absorbance of mitochondrial suspension: A - typical time courses of mitochondrial swelling in standard incubation medium (control, 1) and after the addition of valinomycin (2) and diazoxide (3); B – absorbance changes under the same conditions. The data are mean of 3 independent experiments (n = 3; M ± m; * - P < 0.05). Figure S3. A, B – typical traces showing the time courses of BCECF fluorescence and absorbance in the absence (control) and the presence of 0.5 μM of diazoxide. Other additions are shown on the legends; quinine was added at 0.5 mM, MgCl2 at 1 mM. C, D – the typical changes in PBFI fluorescence in the absence (1, 2) and the presence of DZ (3, 4); * - P < 0.05 (3, 4 vs. 1, 2). Figure S4. A, B: Typical time courses of DCF fluorescence in rat liver mitochondria in standard incubation medium with the additions described in the legends. C: the changes in DCF fluorescence over 4 min of incubation. The data are means of 4 independent experiments (n = 4; M ± m; * - P < 0.05 as compared to controls without DZ). [file 12860_2020_275_MOESM1_ESM.docx]

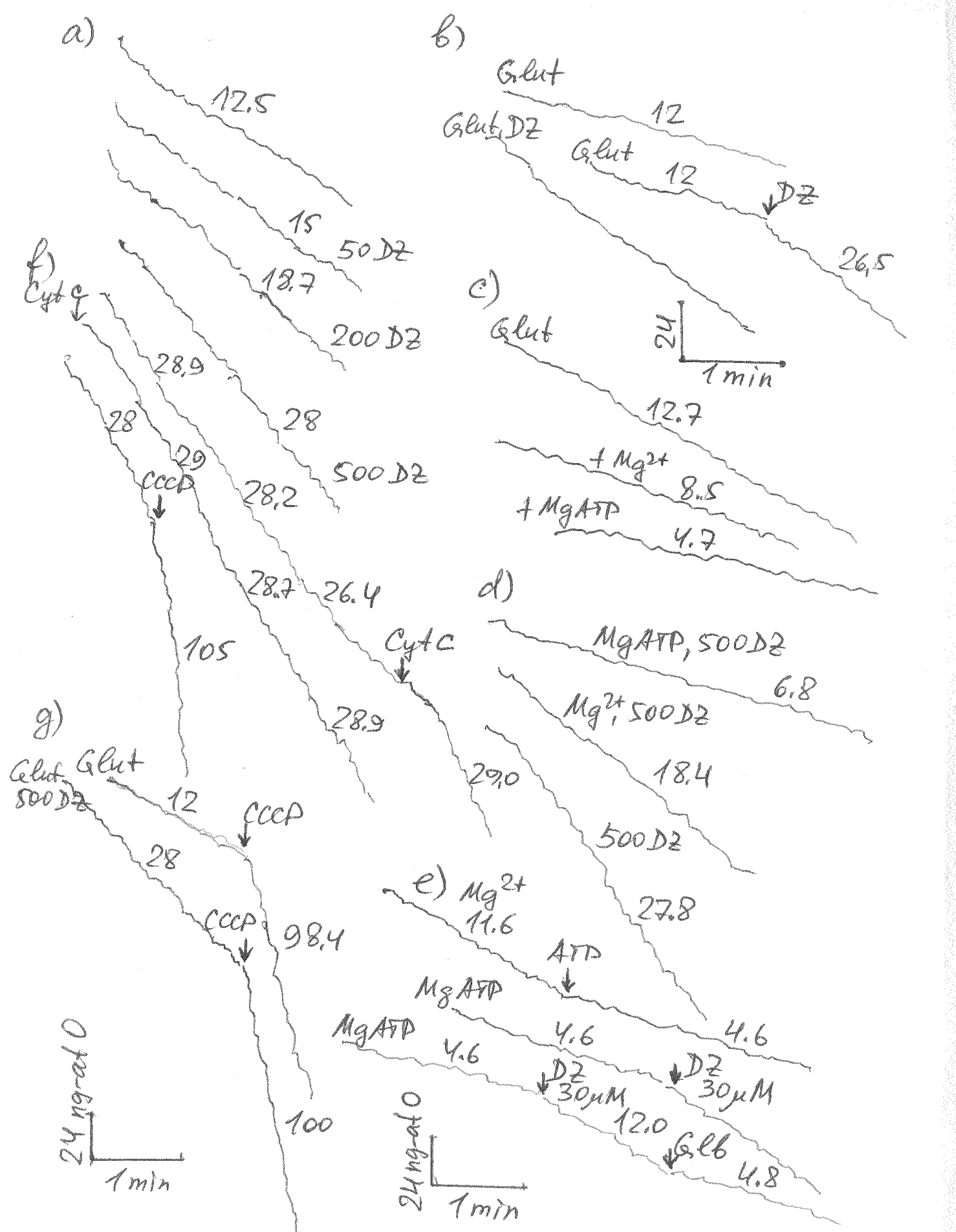


**Fig. 1 Suppl**. Typical polarographic records showing the effect of diazoxide on the glutamate driven respiration of rat liver mitochondria. On the curves are shown the additions to standard incubation medium, the rates of respiration in ng-at. O·min^-1^·mg^-1^, and the sequence of additions.

**Fig. 2 Suppl.** the effect of diazoxide and valinomycin on the absorbance of mitochondrial suspension: A - **t**ypical time courses of mitochondrial swelling in standard incubation medium (control, 1) and after the addition of valinomycin (2) and diazoxide (3); B – absorbance changes under the same conditions. The data are mean of 3 independent experiments (n = 3; M±m; * - P<0.05).

**Fig. 3 Suppl.** A, B – typical traces showing the time courses of BCECF fluorescence and absorbance in the absence (control) and the presence of 0.5 µM of diazoxide. Other additions are shown on the legends; quinine was added at 0.5 mM, MgCl_2_ at 1 mM. C, D – the typical changes in PBFI fluorescence in the absence (1, 2) and the presence of DZ (3, 4); * - P < 0.05 (3, 4 vs. 1, 2).

**Fig. 4 Suppl.** A, B: Typical time courses of DCF fluorescence in rat liver mitochondria in standard incubation medium with the additions described in the legends. C: the changes in DCF fluorescence over 4 min of incubation. The data are means of 4 independent experiments (n = 4; M±m; * - P<0.05 as compared to controls without DZ).
